# A case of humidifier lung; the key diagnosis is detailed medical history taking

**DOI:** 10.1002/rcr2.1200

**Published:** 2023-08-02

**Authors:** Munechika Hara, Yasuaki Yashiro

**Affiliations:** ^1^ Department of Internal Medicine Fujimi‐Kogen Hospital, Fujimi‐Kogen Medical Center Nagano Japan

**Keywords:** dry season, endotoxin, humidifier lung, hypersensitive pneumonitis, medical history taking

## Abstract

A 74‐year‐old woman was admitted with a dry cough and dyspnea that had persisted for 2 weeks at the beginning of winter. Chest computed tomography revealed bilateral diffuse non‐segmental ground‐glass opacities without centrilobular nodules. Bronchoalveolar lavage fluid revealed a marked increase in the lymphocyte ratio. Her condition and chest radiographic findings improved spontaneously after admission. An additional interview conducted after admission revealed that the patient had started using a contaminated humidifier approximately 2 weeks before the onset of symptoms. Thus, the diagnosis of humidifier lung was established. Humidifier lung is a rare phenotype of hypersensitive pneumonitis that often occurs during dry winter when the use of humidifiers increases. Humidifier lung is an important differential diagnosis of bilateral pneumonia during dry winter, and detailed history‐taking regarding the use of humidifiers, assuming a humidifier lung, is crucial for its diagnosis.

## Graphical abstract

Humidifier lung is a phenotype of hypersensitive pneumonitis (HP) caused by inhalation exposure to contaminated humidifier vapours that frequently occur during dry winter. We report a case of humidifier lung that was diagnosed based on its characteristic clinical cause and detailed medical history taking.

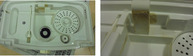

## INTRODUCTION

Humidifier lung is a phenotype of hypersensitive pneumonitis (HP) caused by inhalation exposure to contaminated humidifier vapours that frequently occur during dry winter.^1^ Herein, we report a case of humidifier lung that was diagnosed based on its characteristic clinical cause and detailed medical history taking.

## CASE REPORT

A 74‐year‐old woman was referred to our hospital for dry cough and dyspnea that had persisted since late October. She was prescribed antibiotics by her family physician 2 weeks prior to referral to our hospital; however, the symptoms gradually worsened. The patient was a pharmacist; her medical history included hypertension and allergic rhinitis. She was prescribed antihypertensive medications. The patient had no history of allergies to drugs or food. She had no history of smoking. She resided in a 40‐year‐old wooden house and used quilts.

**FIGURE 1:**
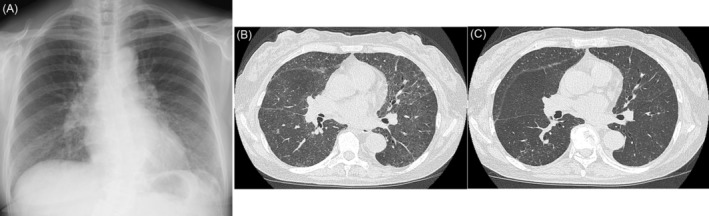
(A) Chest x‐ray showing the ground‐glass opacities in the bilateral lung fields. (B) Thoracic computed tomography (CT) obtained upon admission showing bilateral diffuse nonsegmental ground‐glass opacities. (C) CT obtained 14 days after admission showing improvement of ground‐glass opacities in the bilateral lung fields.

**FIGURE 2:**
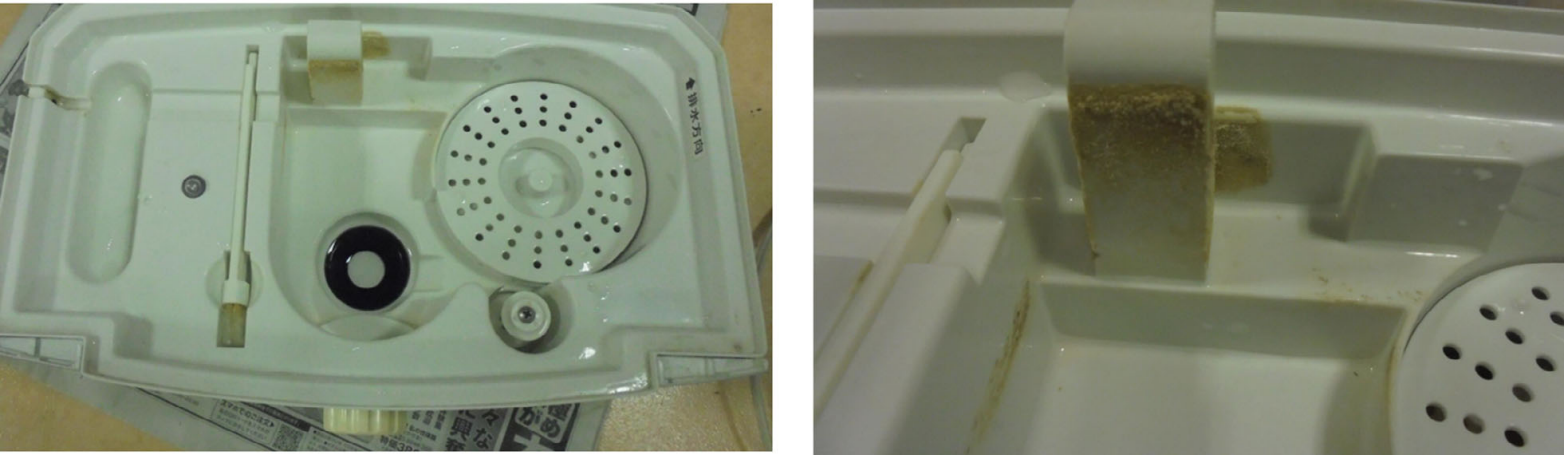
Humidifier used in the patient's home with noticeable contamination.

Physical examination revealed a temperature of 36.4°C and a respiration rate of 18/min with an O₂ saturation of 88% on ambient air. No murmurs were heard, and the lungs were clear on auscultation. A complete blood count revealed a white blood cell count of 8400/μL, with 61.9% segmented neutrophils, 25.6% lymphocytes, and 5.1% eosinophils. Serum chemistry revealed a C‐reactive protein level of 8.4 mg/dL and a Krebs von den Lungen‐6 (KL‐6) level of 536 U/mL, which were slightly elevated. Surfactant protein‐D level was normal at 89.8 ng/mL. Chest radiography upon admission revealed ground‐glass opacities in both lung fields (Figure 1A), and thoracic computed tomography (CT) revealed bilateral diffuse nonsegmental ground‐glass opacities without centrilobular nodules (Figure 1B). Fibre bronchoscopy was performed 4 days after admission, and bronchoalveolar lavage was performed from the left B5 bronchus. The examination of bronchoalveolar lavage fluid (BALF) revealed 3% neutrophils, 75% lymphocytes, and 20% eosinophils. The CD4/CD8 ratio in BALF was 9.48, which was notably elevated. The patient's subjective symptoms, oxygenation, and imaging findings improved spontaneously after hospitalization (Figure 1C). The spontaneous improvement after hospitalization and increased lymphocyte ratio in BALF suggested HP; however, anti‐*Trichosporon asahii* and serum‐specific IgG antibodies against bird antigen were negative. Detailed interviews conducted after hospitalization revealed that the patient had started using a noticeably contaminated humidifier approximately 2 weeks before the onset of symptoms (Figure 2). Although the humidifier was used annually, the patient reported that she had not cleaned it before its use this season. Moreover, she reported that she had never cleaned the humidifier until she was hospitalized. Bacterial cultures of water collected from the humidifier revealed unidentifiable Gram‐negative bacteria and *Bacillus* spp., and the lymphocyte stimulation test was positive. Thus, the patient was diagnosed with a humidifier lung based on her clinical course, radiological findings, and examination findings of BALF and humidifier water. She was discharged from the hospital after prohibiting the use of the humidifier, and her condition has not deteriorated since discharge.

## DISCUSSION

Humidifier lung is a phenotype of HP caused by the inhalation of contaminated humidifier vapours that occurs during dry winters when humidifiers are frequently used.^1^, ^2^

This type of HP was reported for the first time as occupational HP in the 1970s.^3^ Although humidifier lung presents with cough, dyspnea, and fever, as in other phenotypes of HP, it can also present with different clinical features, including lower serum KL‐6 levels, ground‐glass opacities without centrilobular nodules on CT, and high CD4/CD8 ratio in BALF, alveolitis without granuloma, compared with summer‐type HP.^4^, ^5^ Shimoda et al. reported that patients with humidifier lungs showed faster disease progression than those with summer‐type HP.^5^ It is speculated that these differences depend on the pathogenic mechanisms of humidifier lung.^3^ The mechanism of HP is thought to be extrinsic allergic alveolitis with type III or IV granulomas.^1^ In contrast, the pathogenesis of the humidifier lung is thought to involve type III and IV allergies to bacterial and fungal antigens from contaminated humidifiers as well as inhaled endotoxins in humidifier vapour.^2^ Based on the high concentrations of endotoxins found in the humidifier water in many previous reports, it is speculated that Gram‐negative bacteria and their associated endotoxins play an important role in the pathogenesis of humidifier lung.^2^, ^6^, ^7^ Endotoxins are potent agents of lung injury, and endotoxin inhalation could cause pulmonary and systemic symptoms.^8^ Summer‐type HP is caused by continuous inhalation of a relatively small amount of fungal antigen. Patients of humidifier lung develop symptoms after direct exposure to large amounts of antigens, including endotoxin, while using a humidifier.^3^ Alveolar epithelial damage caused by the inhalation of vapours from humidifiers containing large amounts of endotoxin over a relatively short period of time leads to decreased serum KL‐6 levels, lesser centrilobular nodules on CT, and lack of granulomas in humidifier lung.^3^

In the present case, a slight elevation of KL‐6 levels, ground‐glass opacities on chest CT, and a marked increase in the CD4/CD8 ratio in BALF were observed, and the symptoms developed approximately 2 weeks after using the contaminated humidifier. These findings are consistent with those of previous reports.^3^, ^4^ Although endotoxins in the humidifier could not be measured in this case, the fact that Gram‐negative bacteria were cultured from water stored in the humidifier suggests that endotoxins are involved in the pathogenesis. Notably, the eosinophils in the BAL were elevated (20%) in this case, which is atypical for humidifier lung. Although the possibility of acute eosinophilic pneumonia was considered, the marked elevation of the lymphocyte ratio (75%), the predominance of ground‐glass opacities, and the lack of infiltrative shadows are not consistent with the diagnosis of acute eosinophilic pneumonia.^9^ According to the diagnostic criteria for acute eosinophilic pneumonia, the eosinophils in BALF should be ≥25%; the present case did not meet this criterion.^9^ Sakamoto et al. reported an eosinophil ratio of 9.47 ± 19.0% in BALF for humidifier lungs.^4^ Thus, the BAL findings in this patient were consistent with those of humidifier lungs. Although no tissue specimen was obtained in the present case, the CT findings were compatible with HP when the HP diagnostic criteria of the ATS/JRA/ALAT guideline were applied to this case, and the BAL findings showed an increased lymphocyte ratio and obvious exposure to a humidifier. Thus, this case can be diagnosed as a humidifier lung with ‘moderate confidence’ according to the guideline.^1^

Symptoms of fever, cough, dyspnea, and bilateral ground glass opacities on chest CT are seen in HP and other respiratory diseases, including idiopathic interstitial pneumonia, drug‐induced lung injury, and connective tissue disease‐associated interstitial lung disease. Differentiating HP from other respiratory diseases is important, as identification and appropriate avoidance of the causative antigen lead to improvement of prognosis in HP.^10^ Humidifier lungs cannot be diagnosed unless the clinician anticipates the disease and interviews patients regarding the use of humidifiers during the winter season when the frequency of humidifier use increases. In the present case, spontaneous improvement after hospitalization implicated HP, and the addition of a detailed interview regarding humidifier use, the cleaning status, and contamination of the humidifier itself were considered key to the diagnosis of humidifier lung.

Humidifier lung is an important differential diagnosis of pneumonia with ground‐glass opacities in bilateral lung fields during dry winter. Therefore, a detailed history‐taking of humidifier use, assuming a humidifier lung, is of utmost importance for its diagnosis.

## CONFLICT OF INTEREST STATEMENT

None declared.

## ETHICS STATEMENT

The authors declare that appropriate written informed consent was obtained for the publication of this manuscript and accompanying images.

## Data Availability

The data that support the findings of this study are available on request from the corresponding author. The data are not publicly available due to privacy or ethical restrictions.
